# Overexpressed Na_*V*_1.7 Channels Confer Hyperexcitability to *in vitro* Trigeminal Sensory Neurons of Ca_*V*_2.1 Mutant Hemiplegic Migraine Mice

**DOI:** 10.3389/fncel.2021.640709

**Published:** 2021-05-25

**Authors:** Riffat Mehboob, Anna Marchenkova, Arn M. J. M. van den Maagdenberg, Andrea Nistri

**Affiliations:** ^1^Department of Neuroscience, International School for Advanced Studies (SISSA), Trieste, Italy; ^2^Research Unit, Faculty of Allied Health Sciences, University of Lahore, Lahore, Pakistan; ^3^Department of Neurology, Leiden University Medical Center, Leiden, Netherlands; ^4^Department of Human Genetics, University Medical Center, Leiden, Netherlands

**Keywords:** sodium channel, calcium channel, nociception, migraine, purinergic receptors, transgenic mice

## Abstract

Trigeminal sensory neurons of transgenic knock-in (KI) mice expressing the R192Q missense mutation in the α1A subunit of neuronal voltage-gated Ca_*V*_2.1 Ca^2+^ channels, which leads to familial hemiplegic migraine type 1 (FHM1) in patients, exhibit a hyperexcitability phenotype. Here, we show that the expression of Na_*V*_1.7 channels, linked to pain states, is upregulated in KI primary cultures of trigeminal ganglia (TG), as shown by increased expression of its α1 subunit. In the majority of TG neurons, Na_*V*_1.7 channels are co-expressed with ATP-gated P2X3 receptors (P2X3R), which are important nociceptive sensors. Reversing the trigeminal phenotype with selective Ca_*V*_2.1 channel inhibitor ω-agatoxin IVA inhibited Na_*V*_1.7 overexpression. Functionally, KI neurons revealed a TTX-sensitive inward current of larger amplitude that was partially inhibited by selective Na_*V*_1.7 blocker Tp1a. Under current-clamp condition, Tp1a raised the spike threshold of both wild-type (WT) and KI neurons with decreased firing rate in KI cells. Na_*V*_1.7 activator OD1 accelerated firing in WT and KI neurons, a phenomenon blocked by Tp1a. Enhanced expression and function of Na_*V*_1.7 channels in KI TG neurons resulted in higher excitability and facilitated nociceptive signaling. Co-expression of Na_*V*_1.7 channels and P2X3Rs in TGs may explain how hypersensitivity to local stimuli can be relevant to migraine.

## Introduction

Familial hemiplegic migraine type 1 (FHM1) is an autosomal dominant subtype of migraine with aura (IHS, 2013), with similar clinical features (Thomsen et al., 2002)–apart from the hemiparesis–and trigger factors (Hansen et al., 2011) as observed for the common forms of migraine. FHM1 is caused by gain-of-function missense mutations in the pore-forming α1 subunit of neuronal voltage-gated Ca_*V*_2.1 Ca^2+^ channels (Ophoff et al., 1996; Tottene et al., 2002, 2009; Pietrobon, 2013). Introduction of the R192Q missense mutation, previously found in patients with hemiplegic migraine (Ophoff et al., 1996), in the orthologous mouse *Cacna1a* gene resulted in a transgenic FHM1 R192Q knock-in (“R192Q KI”) mouse model (van den Maagdenberg et al., 2004) that expresses key features of migraine pathophysiology and was instrumental in unraveling mechanisms of migraine pathophysiology (Ferrari et al., 2015; Pietrobon and Brennan, 2019). For instance, the mutation causes a gain of function of Ca_*V*_2.1 channels leading to enhanced neurotransmission and cortical hyperexcitability. In addition, an enhanced susceptibility to experimentally induced cortical spreading depolarization (van den Maagdenberg et al., 2004; Eikermann-Haerter et al., 2009; Tottene et al., 2009) and signs of unilateral head pain were observed (Langford et al., 2010; Chanda et al., 2013).

The R192Q mutation also affects the trigeminovascular system, which provides innervation and supplies blood to the orofacial region, asd therefore, is regarded an important contributor and site for migraine pain transduction (Goadsby et al., 2017). For instance, trigeminal ganglia (TG) of R192Q KI mice show a neuroinflammatory profile (Franceschini et al., 2013; Hullugundi et al., 2013), and their TG neurons show upregulated activity of pain-sensing purinergic P2X3 receptors (P2X3Rs) (Nair et al., 2010; Hullugundi et al., 2013), and neuronal hyperexcitability (Fioretti et al., 2011; Hullugundi et al., 2014; Marchenkova et al., 2016a). The hyperexcitability state of KI TG neurons is shown by a lower firing threshold (Fioretti et al., 2011; Hullugundi et al., 2014; Marchenkova et al., 2016a) suggesting that differences in subthreshold conductances, which can facilitate the voltage trajectory to spike discharge, increase the probability to reach the action potential (AP) threshold (Dib-Hajj et al., 2013). Such function can be attributed to various subtypes of subthreshold voltage-gated Na_*V*_ Na^+^ currents (Raman and Bean, 1997; Ogata et al., 2001), some of which are expressed by primary sensory neurons and have been associated with pathological pain conditions (Toledo-Aral et al., 1997; Wood et al., 2004; Dib-Hajj et al., 2010; Habib et al., 2015).

Gain-of-function mutations in α1 subunits of voltage-gated Na^+^ channels Na_*V*_1.7, 1.8 and 1.9 have been linked to pain in humans (Emery et al., 2016; Hoffmann et al., 2018; Shields et al., 2018). Hence there is a rationale to develop selective blockers of Na_*V*_1.7 channels as potential drug targets for the treatment of pain-related diseases (Payandeh and Hackos, 2018). Here we focused on the expression and function of Na_*V*_1.7 channels in wild-type (WT) and KI TG neurons. Both Na^+^ channel types are tightly linked to pain pathways and strongly expressed in sensory neurons (Wood et al., 2004; Dib-Hajj et al., 2010; Habib et al., 2015). Na_*V*_1.7 expression is upregulated in chronic inflammatory (Gould et al., 2000; Black et al., 2004; Strickland et al., 2008), neuropathic and acute pain (Cai et al., 2016).

Like all Na_*V*_1 channels, Na_*V*_1.7 consist of a pore-forming α1 subunit and an auxiliary β subunit (Isom et al., 1992; Ogata and Ohishi, 2002). Both Na^+^ channel types are TTX-sensitive with fast kinetics (Klugbauer et al., 1995; Toledo-Aral et al., 1997). Na_*V*_1.7 current contributes to subthreshold modulatory conductance. Because of their slow voltage-dependent inactivation in response to a small, slow depolarization, Na_*V*_1.7 channels can generate a substantial ramp current, thereby amplifying and facilitating membrane depolarization to reach the spike threshold (Cummins et al., 1998; Herzog et al., 2003). It was shown that rat dorsal root ganglion (DRG) neurons with a Na_*V*_1.7 channel gain-of-function mutation exhibit increased spontaneous and evoked firing (Yang et al., 2016).

We here characterized the expression and function of Na_*V*_1.7 channels in WT and R192Q KI TG neurons to explore their contribution to the enhanced neuronal excitability seen in KI TG neurons, which may have relevance to understanding migraine pathophysiology.

## Materials and Methods

### Mouse Trigeminal Ganglion Cultures

Experiments were performed on TG cultures from homozygous Ca_*V*_2.1 FHM1 R192Q KI and WT mouse littermates. KI mice were obtained from Leiden University Medical Center (van den Maagdenberg et al., 2004) and bred and maintained locally in accordance with the Italian Animal Welfare Act. Genotyping was routinely performed by PCR, as previously reported (Nair et al., 2010). All experimental protocols were in accordance with the EU guidelines (2010/63/EU) and Italian legislation (D.L. 4/3/2014, no. 26), and were approved by the SISSA ethical committee.

For primary culture preparation, TG from P11–P13 mice of both sexes were dissected and enzymatically dissociated for 12 min at 37°C in a mixture containing 0.25 mg/mL trypsin, 1 mg/mL collagenase, and 0.2 mg/mL DNase (Sigma, Milan, Italy) in F-12 medium (Invitrogen, Milan, Italy). Cells were plated on poly-L-lysine-coated petri dishes in F12 medium with 10% fetal calf serum, and used after a 24-h incubation period in accordance with previously described protocols (Simonetti et al., 2006; Hullugundi et al., 2014). For each cell culture series to be used for each experimental protocol (i.e., testing WT, mutant, untreated, treated groups) we harvested six TGs from three mice to yield several culture dishes. For all experiments, KI and WT cultures were used in parallel, at the same time, to allow for direct comparison of experimental data.

### Immunofluorescence

Cells were fixed with 4% paraformaldehyde for 20 min at room temperature (RT) and washed three times with PBS. To reduce non-specific antibody binding, cells were kept in blocking solution (5% fetal bovine serum, 5% of albumin from bovine serum, 0.1% Triton-X 100 in PBS) for 1 h at RT. Cells were then incubated with primary antibodies against Na_*V*_1.7 α1 subunit (mouse anti-SCN9A/PN1, monoclonal, 1:500 in blocking buffer; Acris Antibodies GmbH Cat# AM12054PU-N, RRID:AB_10654661, Herford, Germany) and β-tubulin III (mouse T5076, 1:1,000; Sigma, Milan) was used to specifically mark neurons. Incubation with secondary antibodies (Alexa Fluor 594 goat anti-rabbit, 1:250 and Alexa Fluor 488 goat-anti-mouse 1:250; Invitrogen) was done for 1 h at RT.

For the immunofluorescent staining of Na_*V*_1.7-P2X3R co-expression, cells were incubated with both primary Na_*V*_1.7 α1 subunit antibody (mouse anti-SCN9A/PN1, monoclonal, 1:500 in blocking buffer; Acris) and P2X3R antibody (guinea pig anti-P2X3R, polyclonal, 1:100 in blocking buffer; Neuromics, Edina, MN, United States). Next, an incubation was performed with secondary antibodies (Alexa Fluor 594 goat-anti-mouse, 1:250 and Alexa Fluor 488 goat anti-guinea pig, 1:250; Invitrogen) for 1 h at RT.

Cell nuclei were counterstained with DAPI (1 μg/mL; Sigma) for 5 min. Coverslips were mounted on a microscope slide with Vectashield Mounting Medium (H-1000; Vector Laboratories, Peterborough, United Kingdom). Images from coverslips were acquired using Zeiss Axioscop fluorescence microscope (Zurich, Switzerland). For cell counting, 10 images of each section/experimental condition (100–600 cells) were taken from at least three experiments. Immunoreactive cells and co-expressions were counted using ImageJ manual cell counter plugin^1^. For a number of experiments, Ca_*V*_2.1-specific blocker ω-agatoxin IVA (200 nM; Sigma) was applied overnight (37°C) to cultures (Nair et al., 2010), which were subsequently processed as indicated above.

### Western Blotting

Primary TG cell cultures were washed in PBS, scraped and placed in ice-cold lysis buffer (10 mM *Tris* pH 7.4, 150 mM NaCl, 2 mM EDTA, 2% *n*-octyl-beta-D-glucopyranoside, 100 mM NaF) containing a cocktail of protease inhibitors (Roche Applied Science, Basel, Switzerland). Cells were disrupted mechanically using a syringe and incubated on ice for 20 min. The lysate was then centrifuged (13,000 rpm) at 4°C for 20 min. The supernatant was transferred to a new Eppendorf tube and the same buffer volume was added. For each experiment a minimum of 3 WT (six ganglia and several culture dishes) and 3 KI (six ganglia and several culture dishes) animals were used. A total of 12 ganglia were used for one experiment. One well had a sample from one TG of one animal. Samples were separated on 8% SDS gel, transferred to a PVDF membrane that was subsequently incubated with anti-Na_*V*_1.7 α1 subunit antibody (mouse anti-SCN9A/PN1, monoclonal, 1:1,000; Acris) for 1 h at RT. Mouse anti-rabbit IgG–HRP-conjugated antibody (Jackson ImmunoResearch, Suffolk, United Kingdom) was used as secondary antibody that was incubated for 1 h at RT. Signals were detected with the enhanced chemiluminescence light system ECL (Amersham Biosciences, Piscataway, NJ, United States) and recorded with the digital imaging system Alliance 4.7 (Uvitec, Cambridge, United Kingdom). Quantification of the optical density of the bands was performed with the ImageJ software plug-in. Expression of anti-β-actin antibody (A5441; Sigma) was used as normalization control of gel loading as indicated in the figure legends.

### Electrophysiology

Whole-cell patch-clamp experiments were performed on small- to medium-sized TG neurons thought to be nociceptors (capacitance below 25 pF) after 24 h in culture (Nair et al., 2010; Hullugundi et al., 2013) using a Patch Clamp PC-501A amplifier (Warner Instrument Corporation, Hamden, CT, United States). Cells were continuously superfused at a rate of 2–3 mL/min with physiological solution containing (in mM): 152 NaCl, 5 KCl, 1 MgCl_2_, 2 CaCl_2_, 10 glucose, and 10 HEPES (pH adjusted to 7.4 with NaOH) as previously described (Nair et al., 2010; Hullugundi et al., 2013). Electrophysiological responses were filtered at 2 KHz and acquired by means of a DigiData 1,200 interface and pClamp8.2 software (Molecular Devices, Sunnyvale, CA, United States); series resistance was compensated by 75–90%.

For current-clamp experiments glass pipettes (4–5 MΩ resistance) were filled with the following solution (in mM):125 K-gluconate, 5 KCl, 2 MgCl_2_, 2 Mg_2_ATP_3_, 10 HEPES, and 10 EGTA (pH adjusted to 7.2 with KOH). The liquid junction potential was 14.5 mV, and data were corrected accordingly. Cells were routinely held at −70 mV, apart from the experiments on spontaneous firing when neurons were recorded also at −60 and −50 mV. Depolarizing current pulses (300 ms, 45 pA) were used to elicit cell-specific firing activity from TG neurons, in line with previously used protocols (Hullugundi et al., 2014; Marchenkova et al., 2016a, b). The electrophysiological properties of WT and KI neurons obtained in our lab were published in detail (Hullugundi et al., 2014). It is important to note that neurons can be classified into different categories of spike firing patterns and that this observation is best achieved by using a standard current pulse (300 ms, 45 pA) that elicits a comparable depolarization level. Hence, we used this approach for the experiments.

For voltage-clamp experiments glass pipettes (3–4 MΩ resistance) were filled with a solution containing (in mM): 130 CsCl, 20 HEPES, 1 MgCl_2_, 3 ATP-Mg, 5 EGTA, pH 7.2 adjusted with CsOH. The liquid junction potential was 5 mV, and data were corrected accordingly. Cells were held at −75 mV (after correction for liquid junction potential). Subthreshold inward currents were evoked using a 100-ms square current pulse of 30 mV amplitude (from −75 to −45 mV). External TEA (Sigma; 10 mM) was used to minimize K^+^ currents (Elliott and Elliott, 1993; Yoshimura and de Groat, 1996). TTX (1 μM; Sigma) was used to eliminate the TTX-sensitive part of the Na^+^ inward current (Elliott and Elliott, 1993; Klugbauer et al., 1995; Yoshimura and de Groat, 1996; Tan et al., 2014).

The spider venom toxin ProTx-III [m-TRTX–Tp1a] (Tp1a; Smartox Biotechnology, Saint-Egrève d’Hères, France) (Cardoso et al., 2015) was used to inhibit Na_*V*_1.7 channels selectively. Cells were incubated with 7 nM Tp1a for 30 min to ensure strong block of Na_*V*_1.7 channels (IC_50_ 2.1 nM) (Cardoso et al., 2015), and to minimize block of Na_*V*_1.3 current (IC_50_ 11.5 nM (Cardoso et al., 2015). Data were collected from cells in culture dishes (same culture series), averaged and processed in parallel for comparison. The α-scorpion toxin OD1 (Smartox Biotechnology) was used to activate Na_*V*_1.7 channels (EC_50_ 4.5 nM) (Jalali et al., 2005; Maertens et al., 2006; Motin et al., 2016) OD1 (10 nM) was applied either directly to the recorded neuron using a fast superfusion system (Rapid Solution Changer RSC-200; BioLogic Science Instruments, Claix, France) or via the bathing solution. In the latter tests we used a pair design for each cell with comparison of effects before and after drug application.

### Data Analysis

Data are shown as mean ± standard error of the mean (SEM), with “*n*” indicating the number of analyzed cells (electrophysiology) or experiments (molecular biology). Thus, for immunofluorescence and molecular biology data, each experiment involved three WT and three KI mice that yielded six ganglia per genotype from which culture dishes were prepared and used for testing. Statistical relevance of the sample size was verified with the online sample size analysis software biomath.info^2^ and powerandsamplesize.com^3^ (power = 0.8, type I error rate = 0.05). For statistical analysis the Student’s *t*-test or the Mann–Whitney rank sum test was used after the software-directed choice of parametric or non-parametric data, respectively (Matlab^4^ ; Sigma Plot and Sigma Stat, version 14, Chicago, IL, United States^5^); chi-square test was used to compare proportions. A *p*-value less than 0.05 was accepted as indicative of a statistically significant difference.

When analyzing firing threshold, an algorithm was used that allowed automated threshold detection based on first and second discrete time derivatives of the voltage time-series (Hullugundi et al., 2014; Marchenkova et al., 2016a). The parameters for threshold calculation were determined empirically and kept constant for all analyzed recordings.

## Results

### Expression of Na_*V*_1.7 α1 Subunit in TG Primary Cultures

**FIGURE 1:**
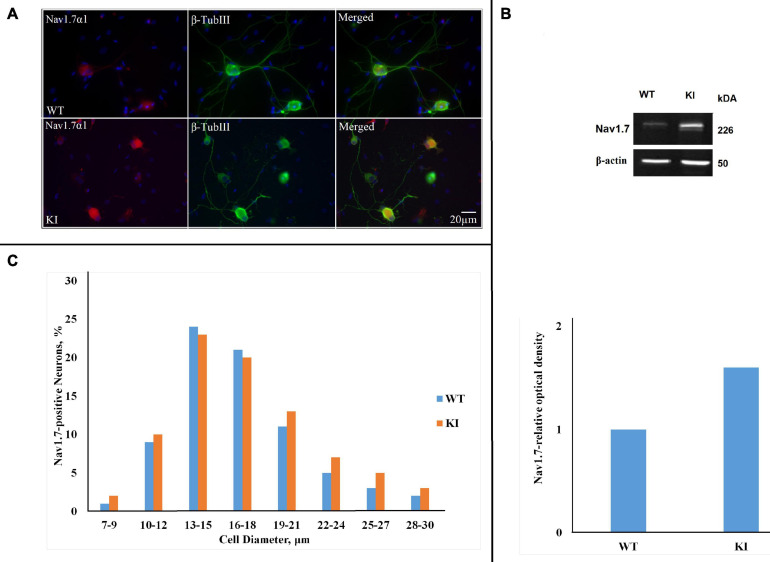
Na_*V*_1.7 α1 subunit expression in wild-type (WT) and R192Q KI TG cultures. **(A)** Representative immunofluorescent examples of Na_*V*_1.7 α1 expression in WT and R192Q KI mouse trigeminal ganglia (TG) cultures. Nuclei are visualized with DAPI (blue); scale bar = 20 μm. Note extensive co-staining of Na_*V*_1.7 α1 (red) and β-tubulin III (green) protein in both WT and KI samples. **(B)** Western blot example showing protein expression of Na_*V*_1.7 α1 in TG from P12 mice. β-actin was used as a loading control. Histograms representing Na_*V*_1.7 α1 relative optical density values for each group (*p = 0.46, Mann-Whitney test; *n* = 8 experiments). Note the significant difference between WT and KI groups. **(C)** The histograms quantify the cell diameter distribution of Na_*V*_1.7 α1 immunofluorescence in different subgroups of WT and KI cultures (*n* = 3 experiments). The percentages of positive neurons for Na_*V*_1.7 were calculated by considering the total number of neurons labeled by β-Tubulin III as standard.

Figure 1A shows immunofluorescent signals of Na_*V*_1.7 α1 subunits. Systematic cell counting indicated that the majority of β-tubulin III-positive cells in WT and KI cultures also expressed Na_*V*_1.7 channels (76 ± 0.1%, *n* = 7 WT cultures, and 84 ± 0.0%, *n* = 7 KI cultures; *p* = 0.016, Mann–Whitney test; see also Figure 1B). Western blot analysis of Na_*V*_1.7 α1 protein showed that the antibody recognized a single band of the expected size (226 kDa) in mouse TG homogenates (Figure 1B). There was, however, a significant difference in the amount of Na_*V*_1.7 protein expressed by WT and KI culture homogenates (Figure 1B; *p* = 0.048, Mann–Whitney test; *n* = 8 experiments). Our data shows that the highest percent of neurons expressing Na_*V*_1.7 were in the 13- to 15- and 16- to 18-μm-diameter groups with similar distribution for WT and KI neurons (Figure 1C).

**FIGURE 2:**
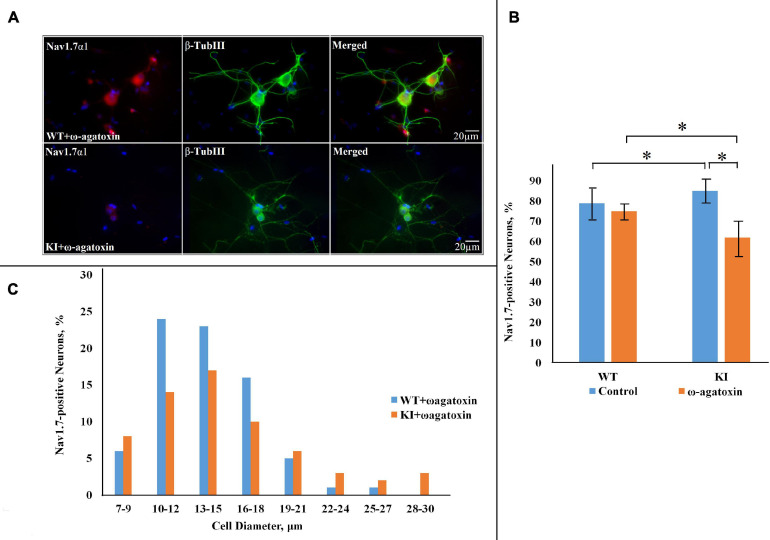
Na_*V*_1.7 α1 subunit expression in WT and R192Q KI TG cultures after pretreatment with ω-agatoxin IVA. **(A)** Representative immunofluorescent examples of Na_*V*_1.7 α1 expression in WT and R192Q KI mouse TG cultures. ω-agatoxin IVA (200 nM, overnight: in this and subsequent Figures the toxin is abbreviated as ω-Agatoxin) was used to specifically block Ca_*V*_2.1 channels (that are mutated in the KI model). Nuclei are visualized with DAPI (blue); scale bar = 20 μm. Note extensive co-staining of Na_*V*_1.7 α1 (red) and β-tubulin III (green) protein in both WT and KI samples. **(B)** Histograms represent the percentage of Na_*V*_1.7-positive cells in WT and KI cultures. Note significant difference between WT and KI control groups (*p = 0.015, Mann–Whitney test; *n* = 7 experiments), and the decrease in Na_*V*_1.7 positive neurons in the KI after application of ω-agatoxin IVA (*p* = 0.035; *n* = 3 experiments). **(C)** The histograms quantify the cell diameter distribution of Na_*V*_1.7 α1 immunofluorescence in different subgroups in WT and KI ω-agatoxin IVA-treated cultures.

Previous studies have demonstrated a lower Ca_*V*_2.1 activation threshold and increased Ca_*V*_2.1 current in several types of KI neurons (van den Maagdenberg et al., 2004; Tottene et al., 2009; Fioretti et al., 2011), and in our TG neuron preparations upregulated P2X3R function (Nair et al., 2010) and a neuroinflammatory ganglion profile (Franceschini et al., 2013). This KI TG phenotype could be reversed by pretreatment with ω-agatoxin IV (200 nM), a selective blocker of Ca_*V*_2.1 channels (Nair et al., 2010; Marchenkova et al., 2016b). Hence, we adopted the same approach to investigate whether Ca_*V*_2.1 inhibition could reverse overexpression of Na_*V*_1.7 channels in KI TG cultures. After overnight treatment with the blocker, Na_*V*_1.7 expression remained unchanged in WT cells (75 ± 0.8%; *p* = 0.732, Mann–Whitney test; *n* = 3 experiments), whereas it was down to 59 ± 0.8% in KI cultures (*p* = 0.003; *n* = 3 experiments) (Figure 2).

**FIGURE 3:**
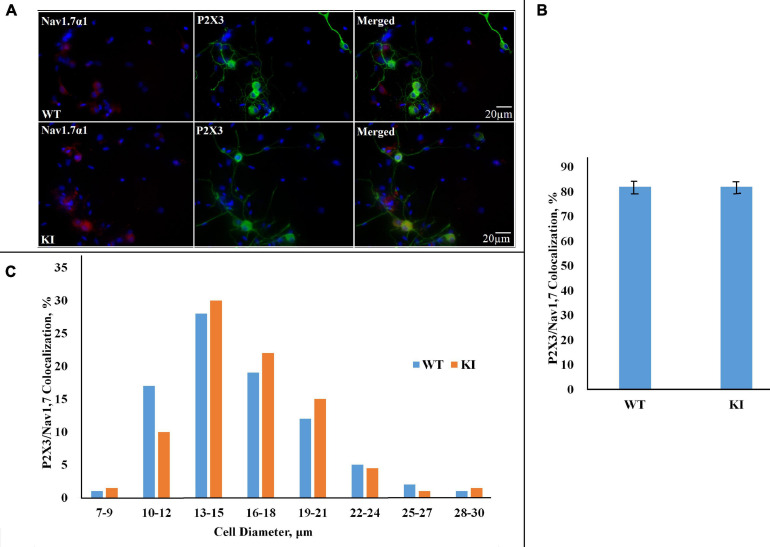
Co-expression of Na_*V*_1.7 α1 subunit and P2X3R in WT and R192Q KI cultures. **(A)** Representative examples of Na_*V*_1.7 α1–P2X3 immunostaining in WT and R192Q KI mouse TG cultures. Nuclei are visualized with DAPI (blue); scale bar = 20 μm. Note the extensive co-staining of Na_*V*_1.7 α1 (red) and P2X3 (green) protein in both WT and KI samples. **(B)** Histograms represent percentage co-expression of Na_*V*_1.7 α1–P2X3 proteins in WT and KI cultures (*p* = 0.95, Mann–Whitney test; *n* = 3 experiments). **(C)** The histograms quantify the cell diameter distribution of Na_*V*_1.7 α1–P2X3R immunofluorescence in different subgroups in WT and KI cultures.

Since P2X3R-expressing neurons are important contributors to trigeminal pain (Wirkner et al., 2005; Burnstock, 2006), we also investigated whether P2X3R immunoreactivity was co-expressed with Na_*V*_1.7 α1 immunopositivity. Figure 3A shows examples of neurons in which both proteins were co-expressed: it is noteworthy that Na_*V*_1.7 α1 immunoreactivity was mainly observed in soma, whereas P2X3R expression was more diffuse in neurons (see Figure 3A for merged images). Histograms show that the majority of Na_*V*_1.7 α1-positive neurons were also positive for P2X3R in both WT and KI cultures (Figure 3B; *p* = 0.95, Mann–Whitney test; *n* = 3 experiments), and the neuronal diameter of the double-positive neurons is in the same range as that of individually stained neurons (Figure 3C).

Observations that a gain-of-function mutation of Na_*V*_1.7 channels led to increased neuronal excitability in rat DRGs (Yang et al., 2016) prompted us to investigate whether the observed increase in Na_*V*_1.7 channel expression in KI TG cultures contributes to the development of the hyperexcitability phenotype typical for a subpopulation of KI TG neurons (Fioretti et al., 2011; Hullugundi et al., 2014; Marchenkova et al., 2016b).

### Na_*V*_1.7 Current in TG Neurons

**FIGURE 4:**
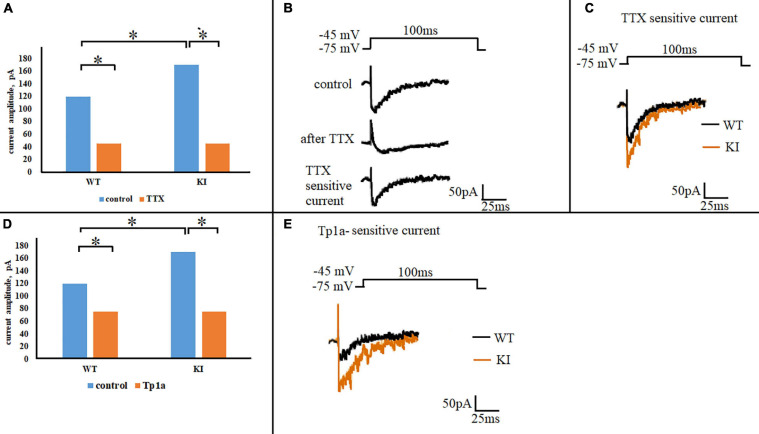
Na_*V*_1.7 current in WT and R192Q KI TG neurons. **(A)** Mean amplitudes of the currents evoked by a square pulse 100 ms depolarizing step from –75 to –45 mV, in WT and R192Q KI TG neurons under control conditions and after application of 1 μM TTX. Note larger control current in KI (*p = 0.039, two-sample Student’s *t*-test). Number of cells in each group: *n* = 29 (WT, control), *n* = 20 (WT, TTX), *n* = 43 (KI, control), *i* = 24 (KI, TTX). **(B)** Representative traces of inward currents recorded from one WT neuron in response to the same stimulation in control and after 1 μM TTX; the trace of TTX-sensitive current was obtained by subtraction of the TTX-resistant current from the control. **(C)** Superimposed representative traces of WT and KI TTX-sensitive currents obtained by subtraction; note larger KI current. **(D)** Histograms represent the calculated mean amplitudes of the currents evoked by the same stimulus (100-ms step from –75 to –45 mV) in control and after Tp1a (7 nM, 30 min). *Indicates statistically significant change (two-sample Student’s *t*-test, *p*-values are given in the text body). Number of cells in each group: *n* = 29 (WT, control), *n* = 28 (WT, Tp1a), *n* = 43 (KI, control), *n* = 38 (KI, Tp1a). **(E)** Superimposed representative traces of WT and KI Tp1a-sensitive (Na_*V*_1.7) currents obtained by subtraction of the TTX-resistant current from the control; note larger Na_*V*_1.7 current in KI.

To study subthreshold Na_*V*_1.7 currents, voltage-clamp experiments were performed with Cs^+^-filled pipettes and extracellular TEA to minimize outward K^+^ currents. A step-change in voltage from holding potential of −75 to −45 mV elicited a transient inward current whose peak was significantly larger in KI compared to WT neurons (Figure 4A, control; *p* = 0.039, two-sample Student’s *t*-test). Application of 1 μM TTX to each neuron significantly reduced WT and KI currents (*p* = 0.2 × 10^–4^ and 0.6 × 10^–5^, respectively, two-sample Student’s *t*-test) (Figure 4A). Residual TTX-resistant current was very similar in WT and KI neurons (*p* = 0.97, two-sample Student’s *t*-test) (Figure 4A). Figure 4B shows an example of control and residual currents recorded from WT TG neurons (similar traces were obtained for KI TG neurons). By subtracting the TTX-resistant current from the total control current, the TTX-sensitive current was obtained that was clearly larger in KI neurons (Figure 4C). On a different batch of cultures we observed that pretreatment of TG neurons with selective Na_*V*_1.7 blocker toxin Tp1a (7 nM, 30 min) (Cardoso et al., 2015) reduced the amplitude of the total inward current in both WT and KI neurons (*p* = 0.026 and 0.028, respectively, two-sample Student’s *t*-test) to the same level (*p* = 0.61, two-sample Student’s *t*-test), as shown in Figure 4D. Subsequent subtraction of Tp1a-resistant current from control current resulted in a Tp1a-sensitive current that was larger in KI neurons (Figure 4E). The total inward current as well as the TTX-sensitive current declined during sustained voltage command to baseline with an analogous time-constant of 19.3 ± 1.2 ms for WT (*n* = 14) and 19.0 ± 1.5 ms for KI (*n* = 11) neurons (*p* = 0.86, two-sample Student’s *t*-test). The similar values between genotypes suggest that there was no systematic change in kinetic properties, but rather a dissimilar expression as indicated by the histochemical observations.

### Blocking Na_*V*_1.7 Channels Reduces Evoked Firing of TG Neurons

**FIGURE 5:**
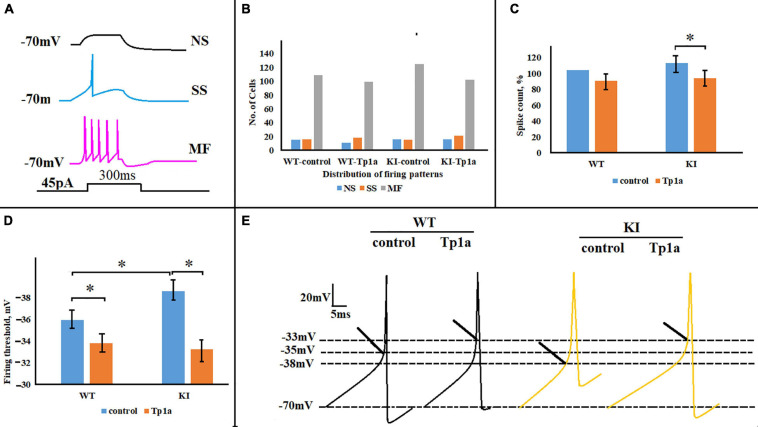
Effects of Tp1a on firing of TG neurons evoked by a 45-pA 300-ms square current pulse. **(A)** Cell-specific firing patterns recorded from three different WT TG neurons in response to the same stimulation. NS, no-spiking cell; SS, single-spike cell; MF, multiple-firing cell. **(B)** Distribution of firing patterns in WT and R192Q KI TG cultures is very similar in control and after application of Tp1a (7 nM, 30 min); chi-square test for proportions. Number of cells in each group for NS, SS, and MF cells, respectively: *n* (WT, control) = 15, 16, 109; *n* (WT, Tp1a) = 11, 18, 99; *n* (KI, control) = 16, 15, 125; *n* (KI, Tp1a) = 16, 21, 102. **(C)** Histograms represent the percent change of the number of spikes, generated by WT and KI neurons in control and after 30 min application of 7 nM Tp1a. *Indicates statistically significant change (Mann–Whitney test, *p*-values are given in the text body). Number of cells in each group: *n* (WT, control) = 109, *n* (WT, Tp1a) = 99, *n* (KI, control) = 133, *n* (KI, Tp1a) = 102. **(D)** Averaged firing threshold of WT and KI neurons in control and after Tp1a (7 nM, 30 min). *Indicates statistically significant change (two-sample Student’s *t*-test, *p*-values are given in the text body). Number of cells is as in **(C)**. Note more negative KI threshold value in control and depolarized WT and KI thresholds after Tp1a application. **(E)** Representative traces of the first generated AP by WT and KI control and Tp1a-treated (7 nM, 30 min) neurons. Arrows indicate AP threshold.

One objective of the study was to investigate whether manipulations aimed at changing the Na_*V*_1.7 conductance could differentially affect the excitability of WT or KI neurons. Hence, we first compared control firing of these two groups of cells by employing the same firing protocol to standardize the spike-generating characteristics of the heterogeneous class of TG neurons. To this end, current-clamp experiments were performed and compared for responses of WT and KI neurons to a depolarizing square current pulse (45 pA, 300 ms) in control solution and after 30 min of 7 nM Tp1a application. This current pulse elicited cell-specific responses from WT and KI neurons, including not-spiking (NS), single-spike (SS), and multiple-firing (MF) cells (see examples in Figure 5A), similar to those previously reported (Hullugundi et al., 2014; Marchenkova et al., 2016a). Blocking Na_*V*_1.7 channels with Tp1a did not significantly affect the distribution of firing patterns in WT and KI cultures (chi-square test for proportions) (Figure 5B). However, significant differences in the number of elicited spikes and firing threshold were detected after Tp1a application (Figures 5C–E). In fact, in KI neurons Tp1a decreased spike numbers/pulse to the level of WT (*p* = 0.008, Mann–Whitney test) (Figure 5C), and, likewise, shifted the threshold value for AP generation (*p* = 5.9 × 10^–9^, two-sample Student’s *t*-test) (Figures 5D,E). Where KI TG neurons have a more negative AP threshold (Figures 5D,E) (see also Marchenkova et al., 2016a), Tp1a application brought WT and KI threshold values to a less negative (i.e., depolarized) level. The effect of Tp1a on WT neurons was similar, although less pronounced with a decreased number of generated spikes (*p* = 0.055, Mann–Whitney test) (Figure 5C) and depolarized firing threshold (*p* = 0.019, two-sample Student’s *t*-test) (Figure 5D).

**FIGURE 6:**
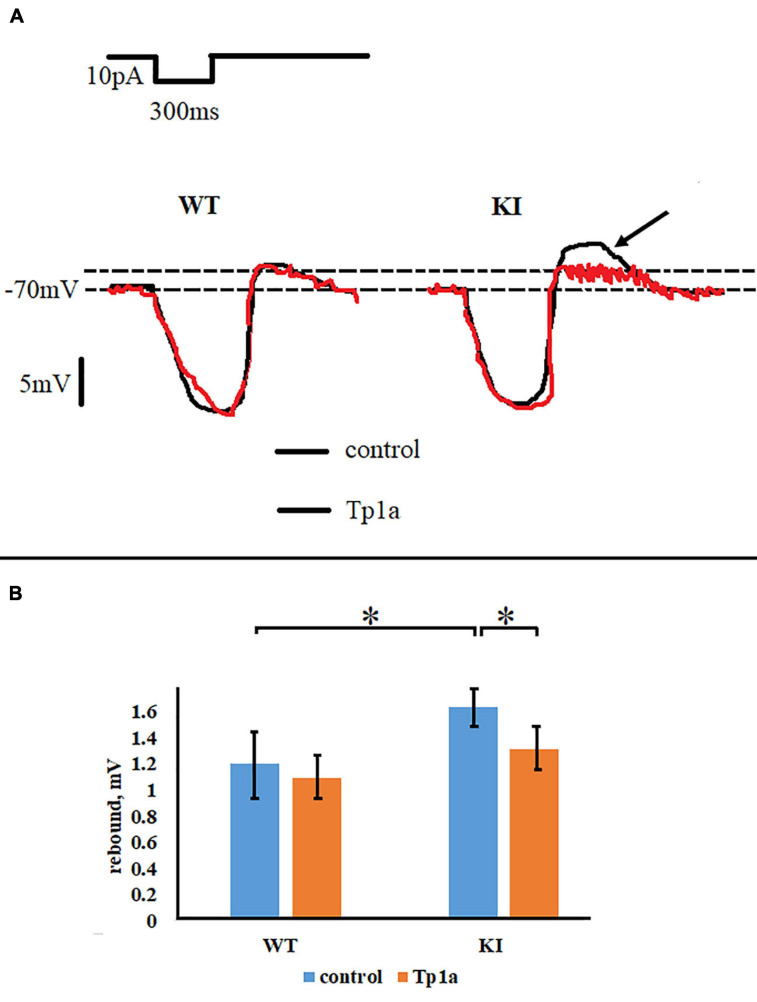
Tp1a reduces rebound effect after a 300-ms hyperpolarizing pulse of 10 pA in R192Q KI TG neurons. **(A)** Representative responses of WT and R192Q KI neurons, recorded in control and after application of Tp1a (7 nM, 30 min). Arrow shows higher rebound in KI which is reduced by Tp1a. **(B)** Histograms represent rebound effect in WT and KI neurons under control conditions and after 30 min application of 7 nM Tp1a. *Indicates statistically significant change (two-sample Student’s *t*-test, *p*-values are given in the text body). Number of cells in each group: *n* = 90 (WT, control), *n* = 86 (WT, Tp1a), *n* = 109 (KI, control), *n* = 96 (KI, Tp1a). Note small but statistically significant difference between WT and KI neurons, and a reduction of the rebound effect in KI after Tp1a application.

In addition to firing properties, Tp1a also changed the size of the rebound effect, observed as a transient depolarization (“off response”) after a 300-ms hyperpolarizing pulse. Figure 6A shows representative recordings of the off response in WT and KI TG neurons, whereas Figure 6B summarizes the averaged values for all cells. The off response in control Krebs solution was statistically larger in KI neurons (*p* = 0.003, two-sample Students *t*-test), but decreased to the WT level (*p* = 0.009, two-sample Student’s *t*-test) after 30 min application of 7 nM Tp1a (Figures 6A,B). Conversely, Tp1a did not affect the off response in WT neurons (*p* = 0.3, two-sample Student’s *t*-test) (Figures 6A,B). These data support the notion that Na_*V*_1.7 channel activation is particularly important for subthreshold behavior of KI neurons.

### Activation of Na_*V*_1.7 Channels Stimulates TG Neuron Firing

**FIGURE 7:**
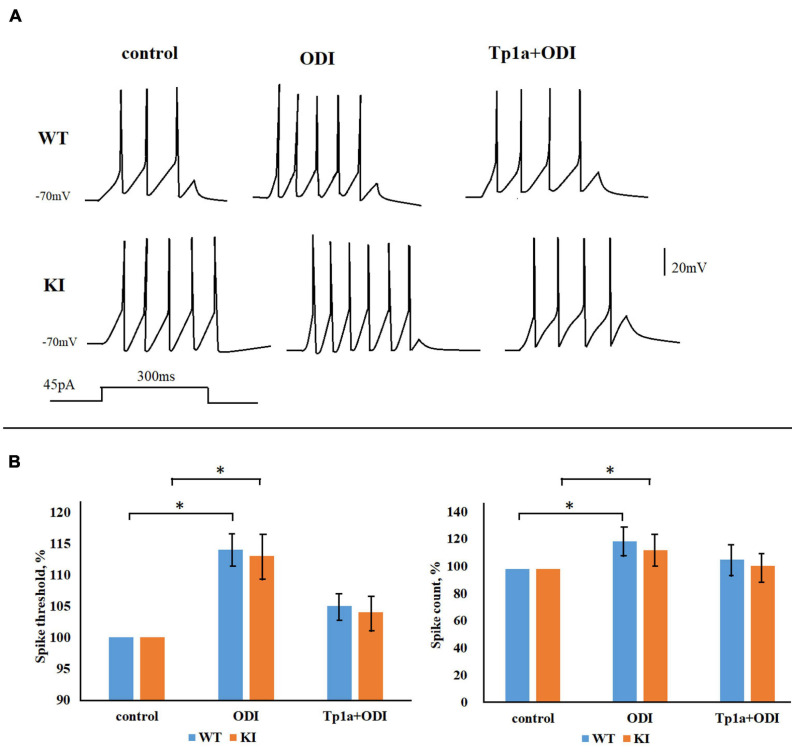
OD1 elevates firing of TG neurons in response to a 45-pA 300-ms square current pulse. **(A)** Representative traces obtained from WT and R192Q KI TG neurons in control and after application of 10 nM OD1 (paired data, recordings from the same cell) or after application of 10 nM OD1 on cells pretreated with Tp1a (7 nM, 30 min; recordings from different cells). **(B)** OD1 application makes the AP threshold more negative and increases spike count both in WT and KI neurons (paired data); *Indicates statistically significant change (Mann-Whitney test, *p*-values are given in the text body). Number of cells in each group: *n* = 37 (WT, control), *n* = 36 (WT, OD1), *i* = 28 (KI, control), *n* = 25 (KI, OD1). The effect of OD1 is decreased when cells were pretreated with Tp1a (7 nM, 30 min); *Indicates statistically significant change (Mann–Whitney test, *p*-values are given in the text body).

Next, we examined the effect of the Na_*V*_1.7 channel activator OD1 (Jalali et al., 2005; Maertens et al., 2006; Motin et al., 2016) on the firing properties of TG neurons. Figure 7A shows representative traces of firing activity, recorded from WT and KI neurons, in response to 45-pA, 300-ms square current pulses in control condition, after application of 10 nM OD1, and when OD1 was applied to cells pretreated with Tp1a (7 nM, 30 min). Figure 7B indicates the average changes in spike threshold and spike count values when OD1 was either directly applied to the control cells (middle bars) or to the cells pretreated with 7 nM Tp1a (right bars). Application of OD1 (10 nM, 10 min) to the recorded cells brought AP threshold to more negative potentials both in WT and KI neurons (Figure 7B shows the difference in%; *p* = 0.005 for WT and *p* = 0.001 for KI, Mann–Whitney test). The effect of OD1 was absent following 30 min pretreatment with 7 nM Tp1a. Application of OD1 also significantly increased the number of generated spikes in response to 45 pA current stimulation (Figure 7B shows the difference in%; *p* = 0.021 for WT and 0.046 for KI, Mann–Whitney test), whereas pretreatment with Tp1a prevented full manifestation of this effect.

### Larger Na_*V*_1.7 Current Underlies Higher Spontaneous Firing Observed in KI Neurons

**FIGURE 8:**
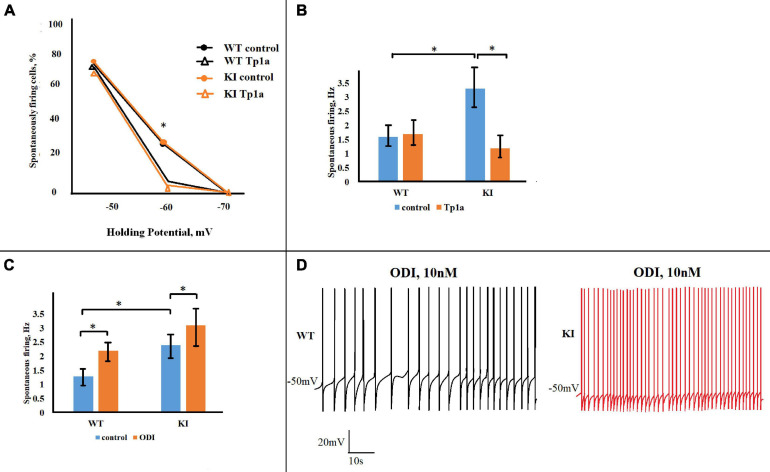
Opposite effects of Tp1a and OD1 on spontaneous firing in TG neurons. **(A)** Diagram shows percentage of firing WT and R192Q KI TG neurons at different holding potentials in control and after Tp1a (7 nM, 30 min). *Indicates statistically significant change (Fisher test for proportions, *p*-values are given in the text body). Number of analyzed neurons at –70, –60, and –50 mV, respectively, are: *n* (WT, control) = 40, 40, 40; *n* (WT, Tp1a) = 32, 32, 31; *n* (KI, control) = 42, 42, 37; *n* (KI, Tp1a) = 33, 33, 33. **(B)** Bar charts represent average firing frequencies of WT and KI neurons in control and after Tp1a (7 nM, 30 min). *Indicates statistically significant change (paired Student’s *t*-test, *p*-values are given in the text body). Number of cells in each group: *n* = 31 (WT, control), *n* = 30 (WT, Tp1a), *n* = 23 (KI, control), *n* = 23 (KI, Tp1a). KI neurons fire with significantly higher frequency, which is reduced to the WT level after Tp1a application. **(C)** Application of 10 nM OD1 (paired data, recordings from the same cell) increases firing frequency of both WT and KI neurons at –50 mV. *Indicates statistically significant change (paired Student’s *t*-test, *p*-values are given in the text). Number of cells in each group: *n* = 17 (WT), *n* = 10 (KI), data are paired. **(D)** Examples of WT and KI neurons, firing at –50 mV before and after application of 10 nM OD1.

The observation that Na_*V*_1.7 activity was acting within the subthreshold voltage range led us to investigate whether this current might influence not only electrically-evoked firing but also spontaneous spike discharges that occur when the neuronal membrane potential fluctuates around (and above) the resting membrane potential. Consequently, we studied the spontaneous generation of APs by WT and KI TG neurons held at −70, −60, and −50 mV. This phenomenon was investigated in each neuron by changing the holding membrane potential within the −70 to −50 mV range and observing the sustained spike discharge (see examples in Figure 8D depicting higher spike discharge of a KI neuron than a WT one). In control solution WT and KI cultures showed the same percent of no active cells at −70 mV and the majority of neurons generating APs at −50 mV (Figure 8A). Figure 8A also shows that while few WT cells could spontaneously fire at −60 mV, more KI neurons did so with or without OD1 application. These results are consistent with the constitutively higher excitability of KI neurons with propensity to fire with smaller depolarizations (Hullugundi et al., 2014). Blocking Na_*V*_1.7 channels with Tp1a (7 nM, 30 min) did not affect the number of firing neurons at −50 mV, but significantly reduced the percent of cells that were active at −60 mV in both WT and KI cultures (*p* = 0.048 for WT and 0.016 for KI, Fisher test) (Figure 8A). It is noteworthy that large differences were detected in spontaneous firing frequency between WT and KI cells as the latter generated, on average, twice as many APs than WT cells at −50 mV (*p* = 0.007, two-sample Student’s *t*-test) (Figure 8B). This difference was abolished by Tp1a, which decreased the firing frequency of KI neurons (*p* = 0.006) to that of WT, without affecting WT neurons (Figure 8B).

Finally, we examined to what extent facilitating the activation of Na_*V*_1.7 channels with OD1 influenced spontaneous firing of WT and KI TG neurons. Consistent with its effect on pulse-evoked responses, OD1 significantly increased spontaneous firing frequency of both WT and KI neurons at −50 mV (*p* = 0.002 for WT and *p* = 0.02 for KI, two-sample Student’s *t*-test) (Figure 8C). It is worth noting that the OD1 effect was proportionally more pronounced in WT neurons (as exemplified in Figure 8D), with their firing frequency being elevated to the level of that in KI neurons.

## Discussion

In this study we investigated the differential expression and function of TTX-sensitive Na_*V*_1.7 channels in trigeminal sensory neurons of WT mice and mice that express the R192Q missense mutation in the α1 subunit of Ca_*V*_2.1 calcium channels that causes FHM1 in patients (Ferrari et al., 2015; Pietrobon and Brennan, 2019). The main finding of our study was a higher expression of Na_*V*_1.7 channels with larger Na_*V*_1.7 current that lowers the threshold for generating APs in KI TG neurons, at least in those of very young mice. We propose that this phenomenon is underlying the hyperexcitability phenotype we observed for KI neurons (Marchenkova et al., 2016a). This phenomenon may contribute to the activation of pain mechanisms, as shown to certain extent in KI mice by Chanda et al. (2013), and perhaps in FHM1 patients. We wish to mention that the hyperexcitability phenotype in KI neurons is not straightforward to interpret, when taking into account data from other laboratories. For instance, Fioretti et al. (2011) have reported a gain of function of Ca_*V*_2.1 channel activity in a subpopulation of adult TG neurons not innervating meninges. Noteworthy, Fioretti et al. employed a different cell culture preparation method that yielded acutely dissociated neurons with minimal processes. Conversely, our procedure produced neurons with long processes (see Figures 1, 2) that may have a distinct chemosensitivity and electroresponsiveness. Our former work based on single cell Ca^2+^ imaging has indicated a wider distribution of Ca_*V*_2.1 activity. In particular, our previous studies have shown that there is similar sensitivity of WT and KI neurons to capsaicin (Hullugundi et al., 2013), and that there are minimal differences in their rheobase and input resistance (Hullugundi et al., 2014). Indeed, KI cells fired more spikes with P2X3 or TRPV1 activation, suggesting a special chemosensitivity phenotype to KI TG neurons.

### Subthreshold Currents in TG Neurons

Neurons, including trigeminal sensory neurons, communicate through APs generated in response to a stimulus (Sunada et al., 1990; Chudler et al., 1991; Coste et al., 2008). The timing and frequency of spikes are essential for encoding information (Eggermont, 1998; Masuda and Aihara, 2007) and, therefore, should be correctly modulated in response to changes in the extracellular milieu. Various conductances are known to play a role in regulating neuronal AP threshold and firing frequency, including currents through various K^+^ (Takeda et al., 2011), low-threshold Ca^2+^ (Kim et al., 2001; Llinas and Steriade, 2006) subthreshold Na^+^ (Waxman et al., 1999; Dib-Hajj et al., 2010; Yang et al., 2016) and hyperpolarization-activated cyclic nucleotide-gated (HCN) (Biel et al., 2009; Cho et al., 2011; He et al., 2014) channels. In the present study, we show that in the FHM1 R192Q KI mouse model TG neuron hyperexcitability phenotype is conferred by increased expression and function of Na_*V*_1.7 Na^+^ channels. One cannot exclude though the potential role of other subthreshold conductances. Afterall, TG neurons also exhibit low-threshold T-type Ca^2+^ (Yoshida and Oka, 1998; Borgland et al., 2001; Ikeda and Matsumoto, 2003; Ross et al., 2009) and *I*_*h*_ (Wells et al., 2007; Cho et al., 2009, 2011) currents, which positively regulate neuronal firing (Kim et al., 2001; Llinas and Steriade, 2006; Cho et al., 2011) and play a role in pathological pain conditions (Emery et al., 2011; Seo et al., 2013). Although at −70 mV a minor fraction of HCN channels might be open as the *I*_*h*_ activation threshold is cell-dependent (Biel et al., 2009; He et al., 2014), rat TG neurons show very little *I*_*h*_ current at this membrane potential (Cho et al., 2011). In addition, *I*_*h*_ current has much slower kinetics than the Na_*V*_1.7 current (Vijayaragavan et al., 2001; Biel et al., 2009; Cho et al., 2011; He et al., 2014) implying their distinct modulation of neuronal firing properties. While the low-threshold T-type Ca^2+^ current has kinetics similar to Na_*V*_1.7 current, this conductance is mostly inactivated at the resting membrane potential (−60 to −70 mV), and requires significant hyperpolarization to remove voltage-dependent channel inactivation (Lovinger and White, 1989; Yoshida and Oka, 1998; Seo et al., 2013) making it an unlikely contributor to firing modulation in our mouse model. Our results accord with previous studies (Shields et al., 2018; Wu et al., 2019; Neff et al., 2020; Siebenga et al., 2020) reporting pharmacological evidence that Na_*v*_1.7 inhibition by its blockers may be involved in relieving pain.

### Higher Expression of Na_*V*_1.7 Channels Contributes to the KI TG Neuronal Hyperexcitability Phenotype

Hyperexcitability of TG neurons in FHM1 R192Q KI mice was previously shown in response to a short current pulse or chemical stimulus (Hullugundi et al., 2014; Marchenkova et al., 2016a). Here we examined genotypic differences between WT and KI TG neurons with respect to neuronal excitability by assessing, under current-clamp condition, spike threshold and firing frequency at different holding potentials. Although no neurons fired at −70 mV, holding them at −60 mV was sufficient to evoke spontaneous firing in a subpopulation of cells. Because Na_*V*_1.7 channel conductance becomes activated at this membrane potential (Yang et al., 2016), its inhibition by Tp1a validated the role of Na_*V*_1.7 in subthreshold properties facilitating the onset of firing. Whereas the percentage of WT and KI neurons generating APs at each test voltage did not differ, KI neurons fired with significantly higher frequency, a difference again abolished by Tp1a. Notably, Tp1a did not affect WT firing frequency, implying that the Na_*V*_1.7 channel current did not play a significant role in regulating repetitive firing of WT neurons under control conditions *per se*. Its role, however, appeared prominent when Na_*V*_1.7 channel expression and function are upregulated in mutated TG neurons. This is consistent with results from a study of DRG neurons that express a gain-of-function Na_*V*_1.7 mutation associated with inherited erythromelalgia and persistent pain states (Yang et al., 2016).

Upregulation of Na_*V*_1.7 channel function in KI TG neurons most likely originated from a higher expression of the channel. Indeed, in KI cultures, the number of Na_*V*_1.7-positive neurons was increased with an apparently higher expression of Na_*V*_1.7 α1 protein in KI than in WT neurons, although immunohistochemistry is at best semi-quantitative. Consistent with these findings, reversing the KI phenotype with Ca_*V*_2.1 blocker ω-agatoxin IVA (Nair et al., 2010; Gnanasekaran et al., 2013) significantly reduced the number of Na_*V*_1.7-positive neurons in KI cultures. There was no detectable difference in Na_*V*_1.7 current kinetics, suggesting no constitutive alteration of KI Na_*V*_1.7 channel properties. The activation of Na_*V*_1.7 channels by OD1 toxin largely increased spike activity in all neurons, albeit more strongly in WT cells. The lower magnitude of the OD1 effect in KI neurons could be attributed to the partially up-regulated properties of the Na_*V*_1.7 channels under basal conditions. The turnover of Na_*V*_1.7 channels is incompletely understood and is likely to be influenced by intracellular modulators including the concentration of free Ca^2+^. We might speculate that the experimentally-observed long-lasting pharmacological inhibition by ω-agatoxin IVA of functionally upregulated Ca_*V*_2.1 channels of KI TG neurons was translated into a downregulation of Na_*V*_1.7 channels. Nonetheless, it is also possible that toxin-dependent partial deprivation of other endogenous substances, such as CGRP and TNFα [upregulated in KI cultures following facilitation of Ca_*V*_2.1 activity (Franceschini et al., 2013; Hullugundi et al., 2013)], regulated Na_*V*_1.7 expression. The mechanisms of Na_*V*_1.7 modulation will require future studies.

### Co-expression of Na_*V*_1.7 Channels and P2X3Rs

ATP-sensitive P2X3Rs are predominantly expressed in sensory ganglion neurons, including TG neurons (Vulchanova et al., 1997; Llewellyn-Smith and Burnstock, 1998) where they control transduction of nociceptive stimuli (Wirkner et al., 2005; Burnstock, 2006). Strong upregulation of P2X3R function and P2X3R-mediated excitability of TG neurons was shown to be associated with the hyperexcitability phenotype of the FHM1 R192Q KI model (Nair et al., 2010; Hullugundi et al., 2013; Marchenkova et al., 2016a,b) further supporting their proposed relevance for triggering migraine pain (Fabbretti, 2013; Yan and Dussor, 2014).

We found extensive co-expression of Na_*V*_1.7 and P2X3R in both WT and KI TG cultures with important implications for TG neuron function. The amount of synthesis/release of natural P2X3R agonist ATP that is known to be enhanced in pathological conditions including migraine (Burnstock et al., 2011) is considered one mechanism leading to the development of neuronal sensitization (Hamilton and McMahon, 2000). In KI TG neurons, the observed co-expression of Na_*V*_1.7 and P2X3R along with increased Na_*V*_1.7 activity can well contribute to the observed P2X3-mediated hyperexcitability, typical for KI TG neurons (Hullugundi et al., 2014; Marchenkova et al., 2016a,b). Taking into account their slow voltage-dependent inactivation (Cummins et al., 1998; Herzog et al., 2003) we propose that Na_*V*_1.7 channels are well-suited to amplify and facilitate depolarization produced by activated P2X3 receptors, leading to higher frequency of spike generation and sensitization of TG sensory neurons.

Thus, neuronal co-expression of Na_*V*_1.7 and P2X3R by small-size neurons within a basal neuroinflammatory milieu comprising of higher ATP levels in the TG of KI mice (Franceschini et al., 2013; Hullugundi et al., 2013) may turn out to be an interesting background for facilitating hypersensitivity to environmental stimuli.

### Possible Relevance to Migraine

The observed hyperexcitability phenotype, namely higher expression of Na_*V*_1.7 channels with larger Na_*V*_1.7 current that lowers the threshold for generating APs in KI TG, was only studied in young mice (P11–P13), and findings should thus not be immediately extrapolated to older mice. In fact, Fioretti et al. (2011), who used mice of the same KI strain studied consequences of the R192Q mutation in 4- to 6-weeks-old animals and showed that the mutation produced a gain-of-function effect (i.e., an increase of Ca^2+^ influx through Ca_*V*_2.1 channels) only in TG neurons that do not innervate the meninges. In TG neurons that innervate the meninges and release CGRP Ca_*V*_2.1 channels seem unaffected by the mutation, suggesting that the R192Q mutation does not alter CGRP release from meninges. Whereas a study by Chan et al. (2019) that investigated the same mutant mice but of 13–14 weeks did not show increased basal nor KCl-stimulated CGRP release in TG either, an increased CGRP release was observed in young mutant mice by Ceruti et al. (2011). Future studies are warranted as they might detect a developmentally-regulated shift in neuronal chemosensitivity. In particular, future *in vivo* studies are needed to translate the *in vitro* observations to *in vivo* application.

At least under our culturing conditions, which involve mild dissociation and mild proteolytic treatment, we found no indication for a change in neuronal Ca_*V*_2.1 expression in dissociated TG neurons versus intact ganglia (Nair et al., 2010).

With respect to the relevance for migraine pathophysiology, the vast majority of small-size TG neurons are highly sensitive to ATP, a very powerful algogenic mediator (Nair et al., 2010). It therefore follows that the large majority of cultured neurons are functional nociceptors. It is helpful to mention that “pure” nociceptors, i.e., responding solely to painful stimuli, are rare because most sensory neurons are polymodal nociceptors, i.e., activated by various sensory stimuli as well as painful ones over a broad receptive field. It should be noted that migraine pain does not originate exclusively from meninges as important trigger areas are, among others, also scalp and neck muscles.

## Conclusion

Na_*V*_1.7 sodium channel antagonism as shown here *in vitro* may be an interesting approach to treat pain-related disorders of the trigeminal system, including migraine. This possibility deserves further exploration in *in vivo* pain models among those that compare effects of antagonists of Na_*V*_1.7 channels.

## Data Availability Statement

The original contributions presented in the study are included in the article/supplementary material, further inquiries can be directed to the corresponding author.

## Ethics Statement

The animal study was reviewed and approved by SISSA Ethical Committee, Trieste, Italy.

## Author Contributions

RM performed the immunofluorescence and Western blotting experiments. AM performed the electrophysiological experiments. AMJMvdM developed the genetic mouse model of hemiplegic migraine. AN supervised the study. All the authors have contributed in the writing and final approval of the manuscript. AMJMvdM and AN also critically reviewed the manuscript.

## Conflict of Interest

The authors declare that the research was conducted in the absence of any commercial or financial relationships that could be construed as a potential conflict of interest.

## Footnotes

1 http://imagej.nih.gov/ij/

2 http://www.biomath.info/

3 http://powerandsamplesize.com

4 https://www.mathworks.com/products/matlab.html

5 https://systatsoftware.com/products/sigmaplot/
